# Engineering T cells with hypoxia-inducible chimeric antigen receptor (HiCAR) for selective tumor killing

**DOI:** 10.1186/s40364-020-00238-9

**Published:** 2020-10-30

**Authors:** Qibin Liao, Huan He, Yunyu Mao, Xiangqing Ding, Xiaoyan Zhang, Jianqing Xu

**Affiliations:** Shanghai Public Health Clinical Center & Institutes of Biomedical Sciences, Fudan University, Shanghai, China

**Keywords:** On-target off-tumor, Hypoxia, Hypoxia-inducible CAR, Hypoxia response element

## Abstract

Chimeric antigen receptor-modified T cells (CAR-T cells) have shown good effects in the treatment of hematologic cancers; however, they may cause on-target off-tumor toxicity because of minimal expression of tumor-associated antigens (TAAs) on normal tissues, particularly in the context of treating solid tumors. Hypoxia is a common hallmark of solid tumors because of the Warburg effect. To minimize side effects, we designed a hypoxia-inducible CAR (HiCAR), which is driven by a hypoxia response element (HRE), and consists of a conventional CAR and an oxygen-dependent degradation domain (ODD) that is actively degraded under normoxia but stabilized under hypoxia. HiCAR-T cells showed enhanced cytotoxicity against tumor cells under hypoxia compared to normoxia in vitro and antitumor efficacy comparable to that of conventional CAR-T cells in vivo. Overall, our study demonstrates the potential of the HiCAR for improving the safety of CAR-T cells to promote the clinical application of CAR-T immunotherapy.

**Supplementary information:**

**Supplementary information** accompanies this paper at 10.1186/s40364-020-00238-9.

To the Editor,

Chimeric antigen receptor-modified T cells (CAR-T cells) have shown strong antitumor activity against hematologic cancers [1, 2], which has led to safety concerns regarding on-target off-tumor toxicity, particularly in normal tissues with low expression levels of tumor-associated antigens (TAAs) that are recognized by CAR-T cells [3, 4]. Recent years have seen the emergence of many strategies to spatiotemporally control CAR-T cell activities through regulating antigen recognition [5, 6], but the application of tumor environmental signals (e.g., acidosis and hypoxia) may represent an attractive strategy to control CAR-T cells. A previous study showed that oxygen-sensitive multichain CAR-T cells are responsive to hypoxia [7], while a simple single-chain hypoxia-inducible CAR-T cells (HiCAR-T cells) could respond to hypoxia within solid tumors is elusive.

We aimed to develop a CAR design that require both antigen recognition and hypoxia sensing to generate optimal T cell activity, thereby reducing on-target off-tumor toxicity. This design, founded on the oxygen-dependent degradation domain (ODD), might be particularly useful for targeting common antigens shared between normal and neoplastic tissues. We first compared the hypoxia-sensitivity of four ODDs, three from hypoxia inducible factor-1α (HIF-1α) and one from activating transcription factor-4 (ATF4) [8], using an mCherry-based reporter system (Additional file 1: Figure S1a). Among the four ODDs, the large HIF-1α ODD conferred the fused reporter gene the lowest baseline expression and the strongest induction under hypoxia condition (Additional file 1: Figure S1b-d). Importantly, the hypoxia-induced mCherry-ODD protein was rapidly downregulated upon return to normoxia (Additional file 1: Figure S1e-g). Consequently, we selected the large HIF-1α ODD to attach to CARs including CD19 CAR, AXL CAR and HER2 CAR for further testing. Consistent with the reporter studies, the resulting CARs, namely HiCAR, exhibited only minimal basal expression in Jurkat T cells under normoxia while their surface expression was profoundly enhanced upon hypoxia (Additional file 2: Figure S2).

These data encouraged us to test whether HiCAR could function in primary T cells. We observed only minimal basal expression under normoxia (5.1% and 43 for CD19-HiCAR, 8.6% and 42 for AXL-HiCAR, and 13.2% and 113 for HER2-HiCAR, respectively), and hypoxia induced a significant increase in CAR expression, with 70.2% and 601 for CD19-HiCAR, 61.9% and 307 for AXL-HiCAR, and 64.9% and 489 for HER2-HiCAR, respectively (Fig. 1a-f). We further characterized the expression dynamics of CD19-HiCAR protein under hypoxic versus normoxic conditions (Additional file 3: Figure S3a & S3d). The t_1/2_ of the induction of HiCAR by hypoxia, HiCAR-T cells was determined to be ~ 12 h, with steady level being reached by 24 h (Additional file 3: Figure S3b-c) and a reduction of 50% after the next 24 h. This dynamics is inconsistent with a previous study [7], and different gene delivery methods in our study may account for such discrepancy. As expected, the expression of conventional CAR counterpart was unaffected by the oxygen concentration. Next, we evaluated the controllability of tumor cell-killing activities of HiCAR by oxygen. Notably, for all three examined HiCAR-T cells, only weak cytolytic activities were observed under normoxia, which was dramatically changed upon hypoxia with selective tumor killing being significantly elevated (Fig. 1g-j). We further evaluated the antitumor efficacy of HiCAR-T cells against solid tumors. CD19 HiCAR-T cells showed slightly reduced antitumor activity against CD19-positive tumors compared to their conventional counterparts (Fig. 1k), while HER2 CAR- and HiCAR-T cells were comparably effective in the treatment of tumors expressing high levels of HER2 (Fig. 1l). It should be noticed that we adopted first and second generation of CAR design respectively for creating CD19 and HER2 HiCAR respectively, indicating ODD fusion as a general approach to achieve oxygen-dependent activation of CAR-T cells.

**Fig. 1:**
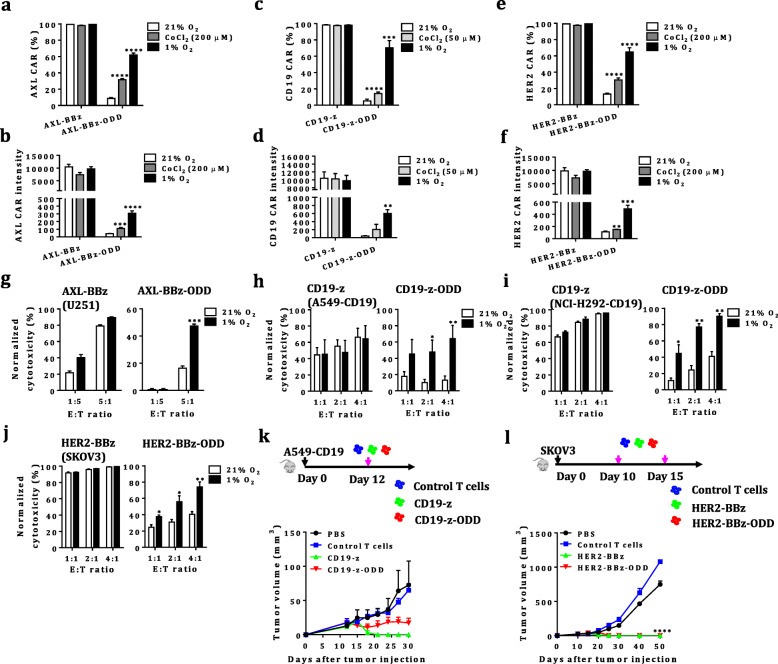
Characterization of HiCAR-T cells. **a**-**f** Effect of oxygen or CoCl_2_ levels on the CAR expression of HiCAR-T cells. AXL (**a**-**b**), CD19 (**c**-**d**) and HER2 (**e-f**). CAR-(e.g., AXL-BBz, CD19-z, HER2-BBz) or HiCAR (e.g., AXL-BBz-ODD, CD19-z-ODD, HER2-BBz-ODD)-engineered T cells were cultured under 21% O_2_, CoCl_2_ or 1% O_2_ for 24 h. **g**-**j** The impact of variations in oxygen levels on the cytolytic properties of HiCAR-T cells toward tumor cells expressing AXL (**g**), CD19 (**h**-**i**) or HER2 (**j**) was evaluated in a luciferase-based killing assay. CAR- or HiCAR-T cells were cocultured with tumor cells for 24 h at indicated E:T ratios. The results are displayed as the mean ± SEM of three independent experiments performed with three healthy donors. Significant differences between normoxia and hypoxia are indicated (*: *p* < 0.05, **: *p* < 0.01, ***: *p* < 0.001, ****: *p* < 0.0001, analyzed using a paired Student’s t-test). **k**-**l** Antitumor efficacy of HiCAR-T cells in vivo. The effects of CAR- or HiCAR-T cells on the growth of CD19-positive A549 tumor cells (**k**). The effects of CAR- or HiCAR-T cells on the growth of SKOV3 tumor cells expressing high levels of HER2. The tumor volumes at different time points are presented as the mean ± SEM (*n* = 5 mice/group), and the significant differences between CAR- or HiCAR-T cells and control T cells are indicated (****: *p* < 0.0001, analyzed using one-way ANOVA) (**l**)

As low CAR expression could result in limited antitumor efficacy [9], we intended to improve the hypoxia-induced HiCAR expression by adding a hypoxia response element (HRE) [10] upstream of EF1α promoter to generate a chimeric HRE-EF1α promoter (chHE). As expected, chHE-HiCARs were observed to exhibit low basal expression under normoxia (Fig. 2a-c). Importantly, significantly enhanced inductions were observed for chHE-HiCAR in relative to HiCAR, as measured by both percentage of positive cells and mean intensity of fluorescence (Fig. 2d-f). Accordingly, when compared to their HiCAR-T equivalents, chHE-HiCAR-T cells showed augmented cytotoxicity against tumors with low levels of TAAs expression both in vitro (Fig. 2h) and in vivo (Fig. 2j), despite with similar antitumor activities against tumors highly expressing TAAs (Fig. 2g and i).

**Fig. 2:**
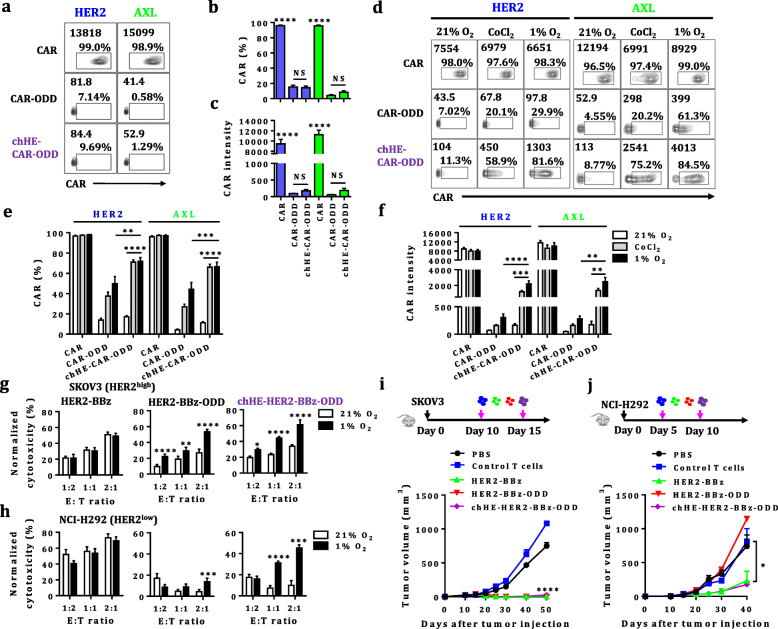
HRE enhances the expression and antitumor potency of HiCAR-T cells. **a**-**c** CAR expression under normoxia. CAR-, HiCAR- or chHE-HiCAR-T cells were cultured under normoxia for 24 h. Representative contour plots of CAR expression under normoxia (**a**); the results are presented as the mean ± SEM of three independent experiments with three healthy donors. Significant differences between HiCAR and chHE-HiCAR are indicated (NS: no significance, **: *p* < 0.01, ***: *p* < 0.001, ****: *p* < 0.0001, analyzed using one-way ANOVA) (**b**-**c**). **d**-**f** The impact of oxygen or CoCl_2_ levels on the CAR expression. The engineered T cells were cultured under 21% O_2_, CoCl_2_ or 1% O_2_ for 24 h. Representative contour plots of CAR expression under normoxia and hypoxia (**d**). These results are displayed as the mean ± SEM of three independent experiments with three healthy donors (**e**-**f**). **g**-**h** The effect of different oxygen levels on the cytotoxic properties of HiCAR-T cells towards tumor cells expressing high levels of HER2 (**g**) or low levels of HER2 (**h**). These results are displayed as the mean ± SEM of three independent experiments with three healthy donors, and significant differences between normoxia and hypoxia are indicated (*: *p* < 0.05, **: *p* < 0.01, ***: *p* < 0.001, ****: *p* < 0.0001, analyzed using a paired Student’s t-test). **i**-**j** The antitumor activity of HiCAR-T cells against tumors in vivo. The effects of CAR-, Hi-CAR or chHE-HiCAR- T cells on the growth of SKOV3 tumor cells (**i**). The impacts of CAR-, HiCAR- or chHE-HiCAR-T cells on the growth of NCI-H292 tumor cells expressing low levels of HER2 (**j**). The tumor volumes at different time points are displayed as the mean ± SEM (*n* = 5 mice/group), and significant differences between CAR-, HiCAR- or chHE-HiCAR-T cells and control T cells are indicated (*: *p* < 0.05, ****: *p* < 0.0001, analyzed using one-way ANOVA)

We further presented an augmented version of HiCAR, chHE-HiCAR, by placing HiCAR under the control of an HRE-EF1α promoter, which directs more robust hypoxia-dependent transcriptional upregulation by recruiting hypoxia-inducible factor 1 (HIF-1) transcriptional activator that is accumulated under hypoxia through increased protein stability (Additional file 4: Figure S4). With its clear advantage over conventional CAR-T cells in the reduction of on-target off-tumor toxicity, HiCAR-T brings new opportunity to immunotherapy for solid tumors.

## Supplementary information

**Additional file 1: Figure S1.** Identification of the ODD that is responsive to the hypoxic environment. **a** Schematic representation of various mCherry-ODD reporter constructs. The hypoxia-inducible reporter system contains the reporter mCherry fused with an ODD derived from ATF4 or HIF-1α, including ATF4 ODD (CAG29349.1, 152–186 aa), the N-terminal ODD of HIF-1α (NP_001521.1, 380–491 aa), the C-terminal ODD of HIF-1α (NP_001521.1, 492–603 aa), and a large ODD of HIF-1α (NP_001521.1, 380–603 aa). **b** mCherry expression in Jurkat T cells transduced with different mCherry-ODD reporter constructs in a normoxic environment. These engineered Jurkat T cells were cultured under normoxia (21% O_2_) for 24 h. The expression of mCherry was assessed by flow cytometry. The results are presented as the mean ± SEM of four independent experiments with technical triplicates, and significant differences in mCherry expression are indicated (*: *p* < 0.05, ***: *p* < 0.001, ****: *p* < 0.0001, analyzed using one-way ANOVA). **c-d** These engineered Jurkat T cells were cultured under normoxia (21% O_2_) or with cobalt chloride (CoCl_2_) for 24 h. The expression of mCherry was assessed by flow cytometry. The percentages (**c**) and mean fluorescence intensities (**d**) of mCherry under chemical hypoxia were normalized to those under normoxia, which are presented as fold-change values. The results are displayed as the mean ± SEM of four independent experiments with technical triplicates, and significant differences in mCherry expression are indicated (**: *p* < 0.01, ****: *p* < 0.0001, analyzed using two-way ANOVA). **e-g** The decay dynamics of mCherry-ODD returned to those under normoxic conditions. Schematic diagram of this decay experiment (**e**). These engineered Jurkat T cells were cultured under chemical hypoxia for 24 h and then exposed to normoxia for another 20 h. The percentages (**f**) and mean fluorescence intensities (**g**) of mCherry under normoxia were normalized to those at 0 h. These results are presented as the mean ± SEM of three independent experiments with technical triplicates, and significant differences are indicated (**: *p* < 0.01, ***: *p* < 0.001, ****: *p* < 0.0001, analyzed using one-way ANOVA).

**Additional file 2: Figure S2.** Characterization of HiCAR in Jurkat T cells. **a-c** The effect of cobalt chloride (CoCl_2_) levels on the surface CAR expression of CD19 HiCAR-T cells. Schematic diagram of CD19 CAR and CD19 HiCAR constructs. The CD19-targeting scFv was fused to the CD8 hinge and transmembrane region, followed by the CD3ζ signaling domain. The ODD was inserted downstream of the CD3ζ signaling domain in the conventional CAR to generate the HiCAR construct (**a**). Jurkat T cells were transduced with either CD19 CAR (CD19-z) or CD19 HiCAR (CD19-z-ODD). The engineered Jurkat T cells were cultured under normoxia (21% O_2_) or chemical hypoxia (CoCl_2_) for 24 h. The surface CAR expression was determined by flow cytometry. The results are displayed as the mean ± SEM of three independent experiments with technical triplicates, and significant differences in CAR expression between normoxia and hypoxia are indicated (**: *p* < 0.01, ****: *p* < 0.0001, analyzed using Student’s t-test) (**b-c**). **d-f** Effect of cobalt chloride (CoCl_2_) or various oxygen levels on the surface CAR expression of AXL HiCAR-T cells. Schematic diagram of the AXL CAR and AXL HiCAR constructs. The AXL-targeting scFv was fused to the CD8 hinge and transmembrane region, followed by the 4-1BB and CD3ζ signaling domains. The ODD was inserted downstream of the CD3ζ signaling domain in the conventional CAR to generate the HiCAR construct (**d**). Jurkat T cells were transduced with either AXL CAR (AXL-BBz) or AXL HiCAR (AXL-BBz-ODD). The engineered Jurkat T cells were cultured under normoxia (21% O_2_), chemical hypoxia (CoCl_2_) and physical hypoxia (1% O_2_) for 24 h. The surface CAR expression was determined by flow cytometry. The results are presented as the mean ± SEM of three independent experiments with technical triplicates, and significant differences in CAR expression between normoxia and hypoxia are indicated (**: *p* < 0.01, ****: *p* < 0.0001, analyzed using Student’s t-test) (**e-f**). **g-j** Impact of cobalt chloride (CoCl_2_) or various oxygen levels on CAR expression in HER2 HiCAR-T cells. Schematic diagram of the HER2 CAR and HER2 HiCAR constructs as described above (**g**). Jurkat T cells were transduced with either HER2 CAR (HER2-BBz) or HER2 HiCAR (HER2-BBz-ODD). The engineered Jurkat T cells were cultured under normoxia (21% O_2_), chemical hypoxia (CoCl_2_) and physical hypoxia (1% O_2_) for 24 h. Total or surface CAR expression was determined by Western blot analysis (**h**) or flow cytometry (**i-j**). The results are presented as the mean ± SEM of three independent experiments with technical triplicates, and significant differences in surface CAR expression between normoxia and hypoxia are indicated (**: *p* < 0.01, ***: *p* < 0.001, ****: *p* < 0.0001, analyzed using Student’s t-test).

**Additional file 3: Figure S3.** Induction and decay kinetics of HiCAR under hypoxia and normoxia. **a** Schematic diagram of the induction experiment. **b-c** CD19 CAR- or CD19 HiCAR-engineered T cells were cultured in normoxic or hypoxic environments for various time points, and the surface expression of CAR was determined using flow cytometry. These results are displayed as the mean ± SEM of three independent experiments with technical triplicates. **d** Schematic diagram of the decay experiment. These engineered T cells were cultured under hypoxia for 24 h and returned to normoxic conditions for 48 h. **e** Time-course analysis of surface CAR expression decay after returning to the normoxic environment. The percentages and intensities were normalized to 100% for CAR or HiCAR at the time when the normoxic environment was set up. The results are displayed as the mean ± SEM of three independent experiments with technical triplicates.

**Additional file 4: Figure S4.** Schematic diagram of the working principle of HiCAR. In the normoxic environment within normal tissues, HiCAR-engineered T cells maintain minimal surface CAR expression using the ubiquitination-proteasome degradation pathway (left panel). Hypoxia is the common hallmark of multiple solid tumors, and increased surface CAR presentation is found on these engineered T cells when they are in the hypoxic environment within solid tumors (middle panel). Insertion of a hypoxia-responsive element (HRE) upstream of the promoter of the lentiviral vector containing the HiCAR construct boosts surface CAR expression to yield enhanced cytolytic potency under hypoxia (right panel).

**Additional file 5.** Detailed materials and methods.

## Data Availability

All supporting data are included in the manuscript and supplemental files. Additional data are available upon reasonable request from the corresponding author.

## Abbreviations

CAR: Chimeric antigen receptor
TAAs: Tumor-associated antigens
HiCAR: Hypoxia-inducible chimeric antigen receptor
HRE: Hypoxia response element
chHE: Chimeric HRE-EF1α promoter
AXL: AXL receptor tyrosine kinase
HER2: Human epidermal growth factor receptor 2
ATCC: American Type Culture Collection
