# Supplementary material for: Engineering T cells with hypoxia-inducible chimeric antigen receptor (HiCAR) for selective tumor killing
Source: Biomark Res. 2020 Oct 30;8:56. doi: 10.1186/s40364-020-00238-9 (PMC7602323; doi:10.1186/s40364-020-00238-9)
Supplement: Supplementary file 1 — Additional file 1: Figure S1. Identification of the ODD that is responsive to the hypoxic environment. a Schematic representation of various mCherry-ODD reporter constructs. The hypoxia-inducible reporter system contains the reporter mCherry fused with an ODD derived from ATF4 or HIF-1α, including ATF4 ODD (CAG29349.1, 152–186 aa), the N-terminal ODD of HIF-1α (NP_001521.1, 380–491 aa), the C-terminal ODD of HIF-1α (NP_001521.1, 492–603 aa), and a large ODD of HIF-1α (NP_001521.1, 380–603 aa). b mCherry expression in Jurkat T cells transduced with different mCherry-ODD reporter constructs in a normoxic environment. These engineered Jurkat T cells were cultured under normoxia (21% O2) for 24 h. The expression of mCherry was assessed by flow cytometry. The results are presented as the mean ± SEM of four independent experiments with technical triplicates, and significant differences in mCherry expression are indicated (*: p < 0.05, ***: p < 0.001, ****: p < 0.0001, analyzed using one-way ANOVA). c-d These engineered Jurkat T cells were cultured under normoxia (21% O2) or with cobalt chloride (CoCl2) for 24 h. The expression of mCherry was assessed by flow cytometry. The percentages (c) and mean fluorescence intensities (d) of mCherry under chemical hypoxia were normalized to those under normoxia, which are presented as fold-change values. The results are displayed as the mean ± SEM of four independent experiments with technical triplicates, and significant differences in mCherry expression are indicated (**: p < 0.01, ****: p < 0.0001, analyzed using two-way ANOVA). e-g The decay dynamics of mCherry-ODD returned to those under normoxic conditions. Schematic diagram of this decay experiment (e). These engineered Jurkat T cells were cultured under chemical hypoxia for 24 h and then exposed to normoxia for another 20 h. The percentages (f) and mean fluorescence intensities (g) of mCherry under normoxia were normalized to those at 0 h. These results are presented as the me [file 40364_2020_238_MOESM1_ESM.docx]

**
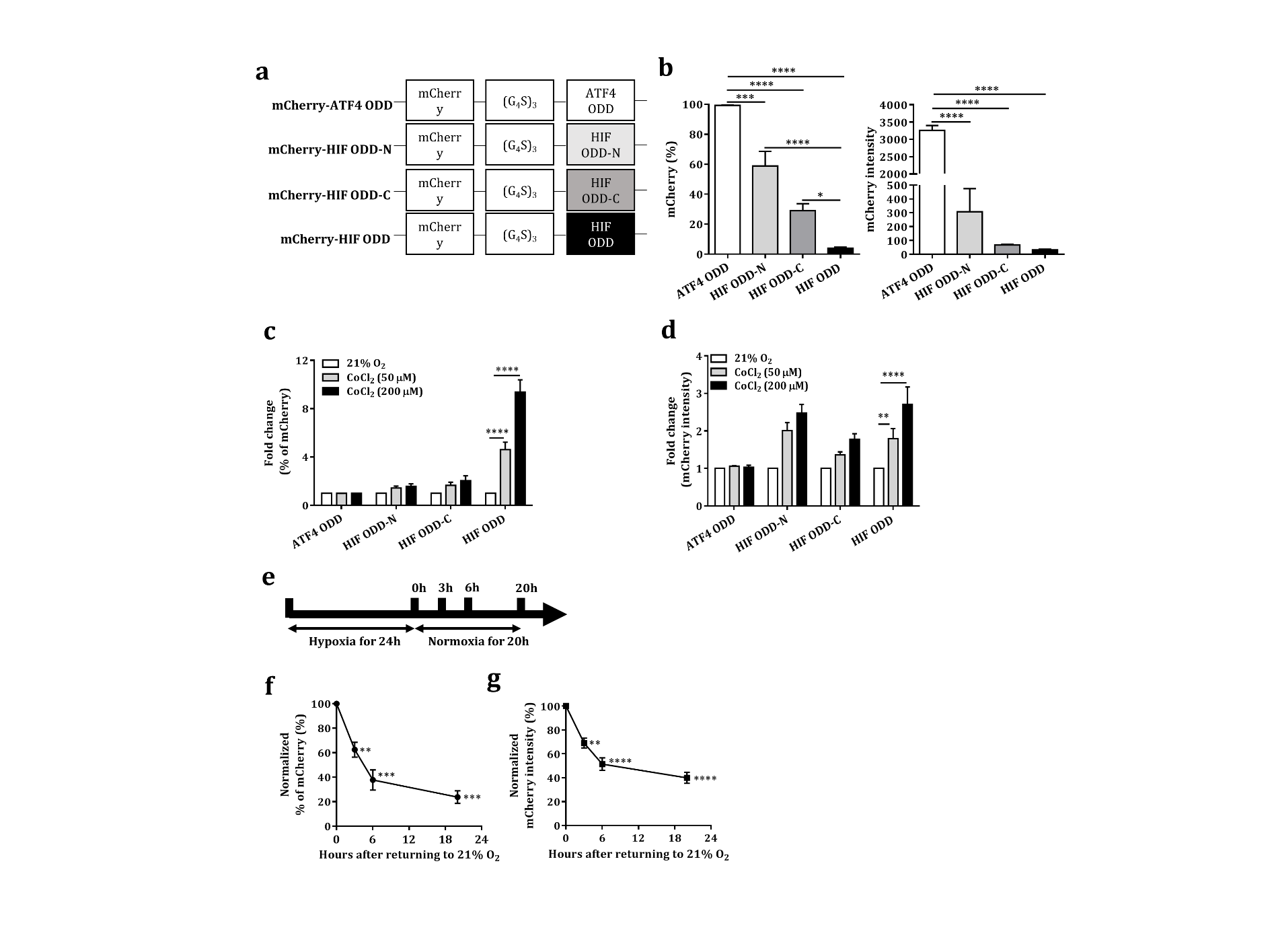
**

**Additional file 1: Figure S1.** Identification of the ODD that is responsive to the hypoxic environment. **a** Schematic representation of various mCherry-ODD reporter constructs. The hypoxia-inducible reporter system contains the reporter mCherry fused with an ODD derived from ATF4 or HIF-1α, including ATF4 ODD (CAG29349.1, 152-186 aa), the N-terminal ODD of HIF-1α (NP_001521.1, 380-491 aa), the C-terminal ODD of HIF-1α (NP_001521.1, 492-603 aa), and a large ODD of HIF-1α (NP_001521.1, 380-603 aa). **b** mCherry expression in Jurkat T cells transduced with different mCherry-ODD reporter constructs in a normoxic environment. These engineered Jurkat T cells were cultured under normoxia (21% O_2_) for 24 h. The expression of mCherry was assessed by flow cytometry. The results are presented as the mean ± SEM of four independent experiments with technical triplicates, and significant differences in mCherry expression are indicated (*: *p* < 0.05, ***: *p* < 0.001, ****: *p* < 0.0001, analyzed using one-way ANOVA). **c-d** These engineered Jurkat T cells were cultured under normoxia (21% O_2_) or with cobalt chloride (CoCl_2_) for 24 h. The expression of mCherry was assessed by flow cytometry. The percentages (**c**) and mean fluorescence intensities (**d**) of mCherry under chemical hypoxia were normalized to those under normoxia, which are presented as fold-change values. The results are displayed as the mean ± SEM of four independent experiments with technical triplicates, and significant differences in mCherry expression are indicated (**: *p* < 0.01, ****: *p* < 0.0001, analyzed using two-way ANOVA). **e-g** The decay dynamics of mCherry-ODD returned to those under normoxic conditions. Schematic diagram of this decay experiment (**e**). These engineered Jurkat T cells were cultured under chemical hypoxia for 24 h and then exposed to normoxia for another 20 h. The percentages (**f**) and mean fluorescence intensities (**g**) of mCherry under normoxia were normalized to those at 0 h. These results are presented as the mean ± SEM of three independent experiments with technical triplicates, and significant differences are indicated (**: *p* < 0.01, ***: *p* < 0.001, ****: *p* < 0.0001, analyzed using one-way ANOVA).
