# Supplementary material for: Engineering T cells with hypoxia-inducible chimeric antigen receptor (HiCAR) for selective tumor killing
Source: Biomark Res. 2020 Oct 30;8:56. doi: 10.1186/s40364-020-00238-9 (PMC7602323; doi:10.1186/s40364-020-00238-9)
Supplement: Supplementary file 2 — Additional file 2: Figure S2. Characterization of HiCAR in Jurkat T cells. a-c The effect of cobalt chloride (CoCl2) levels on the surface CAR expression of CD19 HiCAR-T cells. Schematic diagram of CD19 CAR and CD19 HiCAR constructs. The CD19-targeting scFv was fused to the CD8 hinge and transmembrane region, followed by the CD3ζ signaling domain. The ODD was inserted downstream of the CD3ζ signaling domain in the conventional CAR to generate the HiCAR construct (a). Jurkat T cells were transduced with either CD19 CAR (CD19-z) or CD19 HiCAR (CD19-z-ODD). The engineered Jurkat T cells were cultured under normoxia (21% O2) or chemical hypoxia (CoCl2) for 24 h. The surface CAR expression was determined by flow cytometry. The results are displayed as the mean ± SEM of three independent experiments with technical triplicates, and significant differences in CAR expression between normoxia and hypoxia are indicated (**: p < 0.01, ****: p < 0.0001, analyzed using Student’s t-test) (b-c). d-f Effect of cobalt chloride (CoCl2) or various oxygen levels on the surface CAR expression of AXL HiCAR-T cells. Schematic diagram of the AXL CAR and AXL HiCAR constructs. The AXL-targeting scFv was fused to the CD8 hinge and transmembrane region, followed by the 4-1BB and CD3ζ signaling domains. The ODD was inserted downstream of the CD3ζ signaling domain in the conventional CAR to generate the HiCAR construct (d). Jurkat T cells were transduced with either AXL CAR (AXL-BBz) or AXL HiCAR (AXL-BBz-ODD). The engineered Jurkat T cells were cultured under normoxia (21% O2), chemical hypoxia (CoCl2) and physical hypoxia (1% O2) for 24 h. The surface CAR expression was determined by flow cytometry. The results are presented as the mean ± SEM of three independent experiments with technical triplicates, and significant differences in CAR expression between normoxia and hypoxia are indicated (**: p < 0.01, ****: p < 0.0001, analyzed using Student’s t-test) (e-f). g-j Impact of cobalt chloride (Co [file 40364_2020_238_MOESM2_ESM.docx]

**
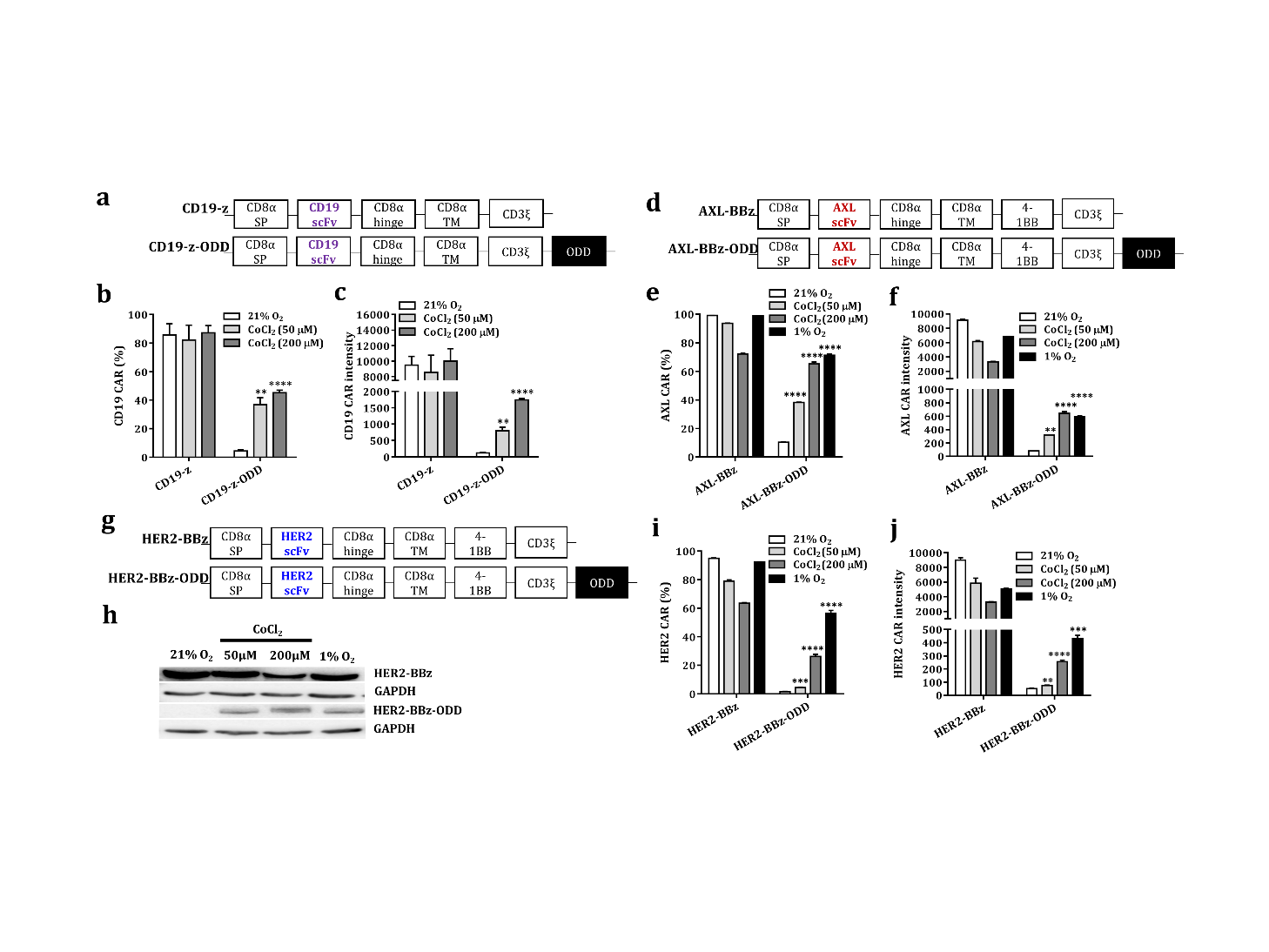
**

**Additional file 2: Figure S2.** Characterization of HiCAR in Jurkat T cells. **a-c** The effect of cobalt chloride (CoCl_2_) levels on the surface CAR expression of CD19 HiCAR-T cells. Schematic diagram of CD19 CAR and CD19 HiCAR constructs. The CD19-targeting scFv was fused to the CD8 hinge and transmembrane region, followed by the CD3ζ signaling domain. The ODD was inserted downstream of the CD3ζ signaling domain in the conventional CAR to generate the HiCAR construct (**a**). Jurkat T cells were transduced with either CD19 CAR (CD19-z) or CD19 HiCAR (CD19-z-ODD). The engineered Jurkat T cells were cultured under normoxia (21% O_2_) or chemical hypoxia (CoCl_2_) for 24 h. The surface CAR expression was determined by flow cytometry. The results are displayed as the mean ± SEM of three independent experiments with technical triplicates, and significant differences in CAR expression between normoxia and hypoxia are indicated (**: *p* < 0.01, ****: *p* < 0.0001, analyzed using Student’s t-test) (**b-c**). **d-f** Effect of cobalt chloride (CoCl_2_) or various oxygen levels on the surface CAR expression of AXL HiCAR-T cells. Schematic diagram of the AXL CAR and AXL HiCAR constructs. The AXL-targeting scFv was fused to the CD8 hinge and transmembrane region, followed by the 4-1BB and CD3ζ signaling domains. The ODD was inserted downstream of the CD3ζ signaling domain in the conventional CAR to generate the HiCAR construct (**d**). Jurkat T cells were transduced with either AXL CAR (AXL-BBz) or AXL HiCAR (AXL-BBz-ODD). The engineered Jurkat T cells were cultured under normoxia (21% O_2_), chemical hypoxia (CoCl_2_) and physical hypoxia (1% O_2_) for 24 h. The surface CAR expression was determined by flow cytometry. The results are presented as the mean ± SEM of three independent experiments with technical triplicates, and significant differences in CAR expression between normoxia and hypoxia are indicated (**: *p* < 0.01, ****: *p* < 0.0001, analyzed using Student’s t-test) (**e-f**). **g-j** Impact of cobalt chloride (CoCl_2_) or various oxygen levels on CAR expression in HER2 HiCAR-T cells. Schematic diagram of the HER2 CAR and HER2 HiCAR constructs as described above (**g**). Jurkat T cells were transduced with either HER2 CAR (HER2-BBz) or HER2 HiCAR (HER2-BBz-ODD). The engineered Jurkat T cells were cultured under normoxia (21% O_2_), chemical hypoxia (CoCl_2_) and physical hypoxia (1% O_2_) for 24 h. Total or surface CAR expression was determined by Western blot analysis (**h**) or flow cytometry (**i-j**). The results are presented as the mean ± SEM of three independent experiments with technical triplicates, and significant differences in surface CAR expression between normoxia and hypoxia are indicated (**: *p* < 0.01, ***: *p* < 0.001, ****: *p* < 0.0001, analyzed using Student’s t-test).
