# Supplementary material for: Engineering T cells with hypoxia-inducible chimeric antigen receptor (HiCAR) for selective tumor killing
Source: Biomark Res. 2020 Oct 30;8:56. doi: 10.1186/s40364-020-00238-9 (PMC7602323; doi:10.1186/s40364-020-00238-9)
Supplement: Supplementary file 3 — Additional file 3: Figure S3. Induction and decay kinetics of HiCAR under hypoxia and normoxia. a Schematic diagram of the induction experiment. b-c CD19 CAR- or CD19 HiCAR-engineered T cells were cultured in normoxic or hypoxic environments for various time points, and the surface expression of CAR was determined using flow cytometry. These results are displayed as the mean ± SEM of three independent experiments with technical triplicates. d Schematic diagram of the decay experiment. These engineered T cells were cultured under hypoxia for 24 h and returned to normoxic conditions for 48 h. e Time-course analysis of surface CAR expression decay after returning to the normoxic environment. The percentages and intensities were normalized to 100% for CAR or HiCAR at the time when the normoxic environment was set up. The results are displayed as the mean ± SEM of three independent experiments with technical triplicates. [file 40364_2020_238_MOESM3_ESM.docx]

**
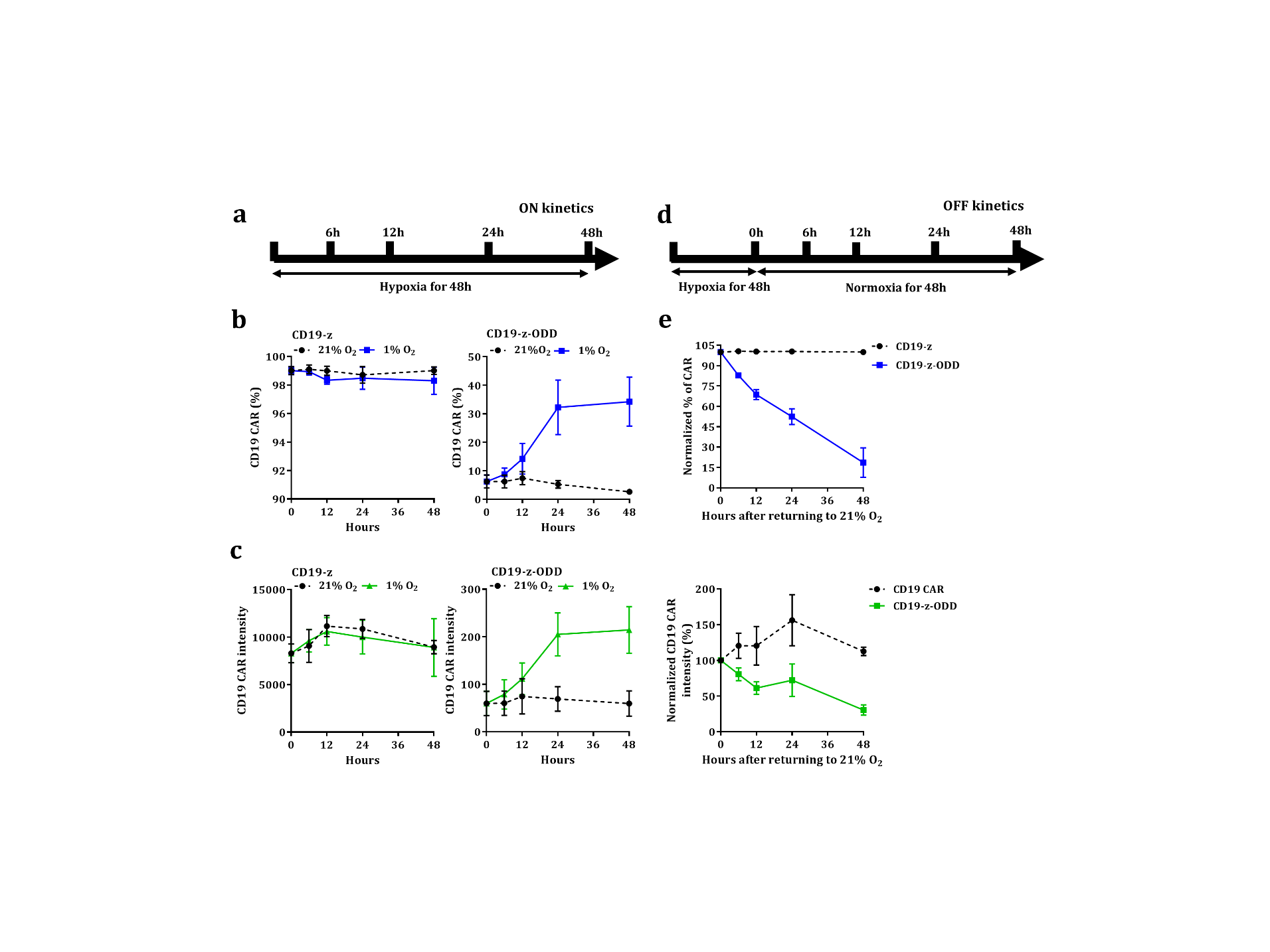
**

**Additional file 3: Figure S3.** Induction and decay kinetics of HiCAR under hypoxia and normoxia. **a** Schematic diagram of the induction experiment. **b-c** CD19 CAR- or CD19 HiCAR-engineered T cells were cultured in normoxic or hypoxic environments for various time points, and the surface expression of CAR was determined using flow cytometry. These results are displayed as the mean ± SEM of three independent experiments with technical triplicates. **d** Schematic diagram of the decay experiment. These engineered T cells were cultured under hypoxia for 24 h and returned to normoxic conditions for 48 h. **e** Time-course analysis of surface CAR expression decay after returning to the normoxic environment. The percentages and intensities were normalized to 100% for CAR or HiCAR at the time when the normoxic environment was set up. The results are displayed as the mean ± SEM of three independent experiments with technical triplicates.
