# Supplementary material for: Engineering T cells with hypoxia-inducible chimeric antigen receptor (HiCAR) for selective tumor killing
Source: Biomark Res. 2020 Oct 30;8:56. doi: 10.1186/s40364-020-00238-9 (PMC7602323; doi:10.1186/s40364-020-00238-9)
Supplement: Supplementary file 4 — Additional file 4: Figure S4. Schematic diagram of the working principle of HiCAR. In the normoxic environment within normal tissues, HiCAR-engineered T cells maintain minimal surface CAR expression using the ubiquitination-proteasome degradation pathway (left panel). Hypoxia is the common hallmark of multiple solid tumors, and increased surface CAR presentation is found on these engineered T cells when they are in the hypoxic environment within solid tumors (middle panel). Insertion of a hypoxia-responsive element (HRE) upstream of the promoter of the lentiviral vector containing the HiCAR construct boosts surface CAR expression to yield enhanced cytolytic potency under hypoxia (right panel). [file 40364_2020_238_MOESM4_ESM.docx]

**
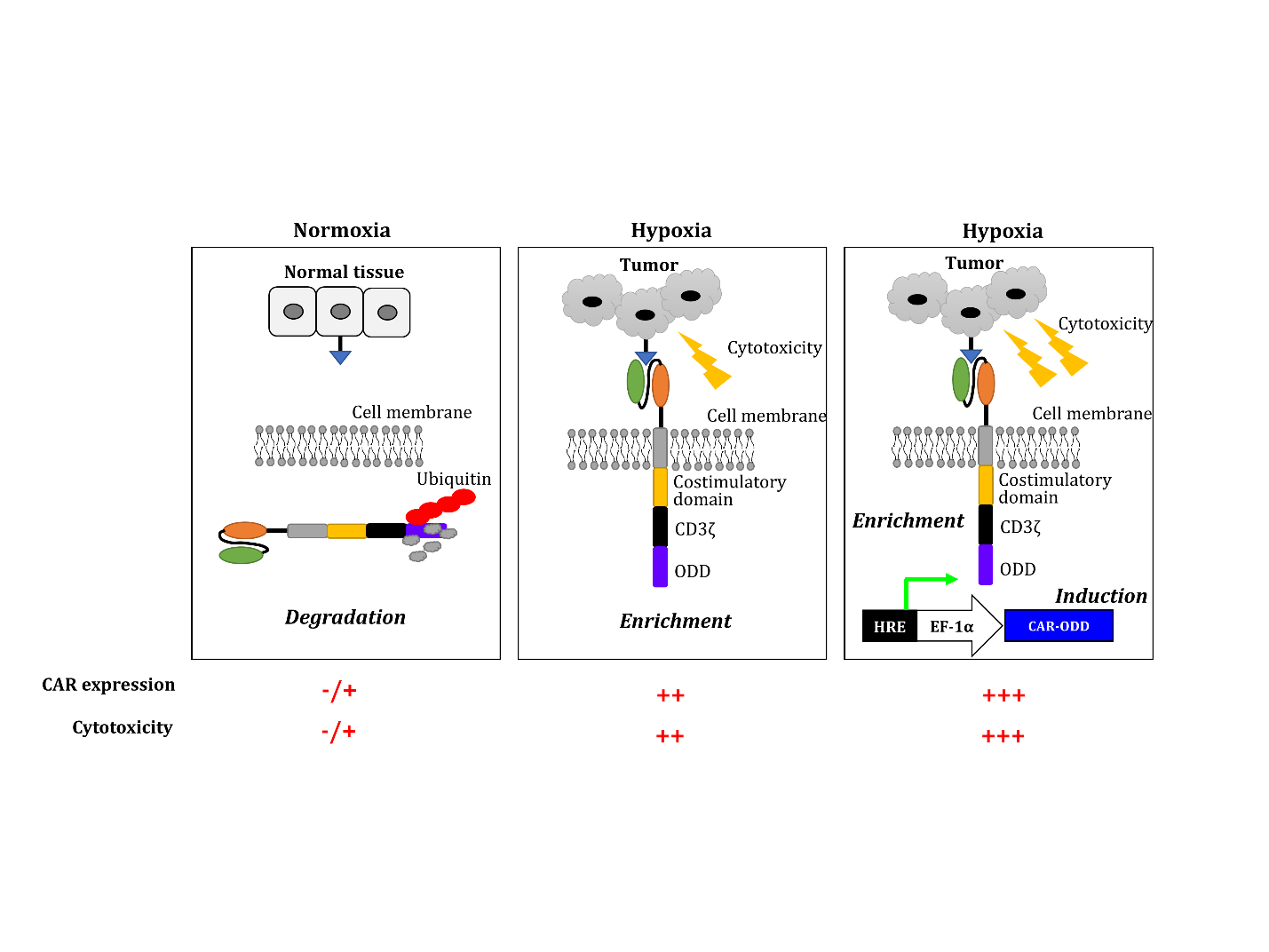
**

**Additional file 4: Figure S4.** Schematic diagram of the working principle of HiCAR. In the normoxic environment within normal tissues, HiCAR-engineered T cells maintain minimal surface CAR expression using the ubiquitination-proteasome degradation pathway (**left panel**). Hypoxia is the common hallmark of multiple solid tumors, and increased surface CAR presentation is found on these engineered T cells when they are in the hypoxic environment within solid tumors (**middle panel**). Insertion of a hypoxia-responsive element (HRE) upstream of the promoter of the lentiviral vector containing the HiCAR construct boosts surface CAR expression to yield enhanced cytolytic potency under hypoxia (**right panel**).
