# Supplementary material for: Engineering T cells with hypoxia-inducible chimeric antigen receptor (HiCAR) for selective tumor killing
Source: Biomark Res. 2020 Oct 30;8:56. doi: 10.1186/s40364-020-00238-9 (PMC7602323; doi:10.1186/s40364-020-00238-9)
Supplement: Supplementary file 5 — Additional file 5. Detailed materials and methods. [file 40364_2020_238_MOESM5_ESM.docx]

**Additional file 5: Detailed materials and methods**

***Cell lines***

The following cell lines were used: HEK293T (embryonic kidney cell line, ATCC #CRL-3216), U373-MG (brain glioblastoma cell line, ATCC #HTB-17), A549 (lung cancer cell line, ATCC #CCL-185), NCI-H292 (lung cancer cell line, ATCC #CRL-1848), SKOV3 (ovarian cancer cell line, ATCC #HTB-77) and Jurkat T cells, Clone E6-1 (acute T cell leukemia, ATCC #TIB-152). All cell lines were purchased from American Type Culture Collection (ATCC) and maintained in RPMI 1640 medium (Corning #10-040-CVR) supplemented with 10% FBS (BI #04-001-1acs) and 1% penicillin-streptomycin (Corning #30-002-CI), except for the HEK293T cells, which were cultured in DMEM (Corning #10-013-CV) supplemented with 10% FBS and 1% penicillin-streptomycin. The firefly luciferase gene was delivered into tumor cells to generate U251-Luc, A549-Luc, NCI-H292-Luc and SKOV3-Luc cells using lentiviral particles. Furthermore, human CD19 was also lentivirally delivered into the A549-Luc and NCI-H292-Luc cell lines to produce versions that had high, stable expression of CD19, e.g., A549-Luc-CD19 and NCI-H292-Luc-CD19. These cells were maintained in a humidified atmosphere containing 5% CO_2_ at 37°C.

***Lentiviral vector designs***

The hypoxia-inducible reporter system contains an mCherry reporter fused with an ODD derived from ATF4 or HIF-1α, including the ATF4 ODD (CAG29349.1, 152-186 aa), the N-terminal ODD of HIF-1α (NP_001521.1, 380-491 aa), the C-terminal ODD of HIF-1α (NP_001521.1, 492-603 aa), and a large ODD of HIF-1α (NP_001521.1, 380-603 aa). All hypoxia-inducible reporter system constructs were cloned into the lentiviral vector pHAGE_EF1α_MCS_IRES_ZsGreen. Human CD19 hypoxia-inducible chimeric antigen receptor (CD19 HiCAR) contains an N-terminal CD8α signal peptide (MALPVTALLLPLALLLHAARP) for membrane targeting, a FLAG tag (DYKDDDDK) for CAR surface detection, a single-chain variable fragment (scFv) of human CD19 (FMC63 clone), a CD8α hinge region, a CD8α transmembrane domain, a CD3ξ signaling domain, and a large ODD (NP_001521.1, 380-603 aa). Human HER2/AXL HiCAR also contains an N-terminal CD8α signal peptide (MALPVTALLLPLALLLHAARP) for membrane targeting, a FLAG tag (DYKDDDDK) for surface detection, human HER2/AXL-targeting scFv, a CD8 hinge and transmembrane domain, a costimulatory domain of 4-1BB, a CD3ξ signaling domain, and a large ODD (NP_001521.1, 380-603 aa). All the CAR constructs were also cloned into another self-inactivating lentiviral vector ABpCCL_sin_EF1α_MCS_P2A_EGFP. All the constructs were synthetized by Generay (Shanghai Generay Biotech Co., Ltd.).

***Primary human T cell isolation and culture***

Primary human peripheral blood mononuclear cells (PBMCs) were isolated from healthy donor blood after apheresis by the Ficoll-Paque density gradient approach. Blood was collected from healthy donors, as approved by the Shanghai Public Health Clinic Center Review Board. PBMCs were cryopreserved in fetal bovine serum (BI) with 10% DMSO. After thawing, PBMCs were cultured in human T cell growth medium consisting of Lymphocyte Serum-Free Medium (Lonza #BE02-060F), IL-7 (5 ng/mL, R&D systems #P13232), IL-15 (10 ng/mL, R&D systems #P40933) and IL-21 (30 ng/mL, Novoprotein #GMP-CC45) for subsequent experiments.

***Lentivirus production***

Transient lentiviral supernatant was produced as described below. Lentiviral particles were prepared by transient transfection of HEK293T cells using TurboFect transfection reagent (Thermo Scientific #R0531). HEK293T cells cultured in 10-cm tissue culture dishes were transfected with 3 μg of the lentiviral backbone plasmid, along with 3 μg of the VSV-G envelope plasmid PMD2.G (Addgene #12259) and 9 μg of the packaging plasmid psPAX2 (Addgene #12260) encoding gag-pol. The lentiviral supernatant was harvested 48 h post-transfection and filtered through a 0.45-μm filter (PALL #4614). Lentiviral particles were concentrated by ultracentrifugation for 2 h at 28000 rpm with a Beckman SW28 rotor (Beckman) for further use.

***Primary T cell transduction and expansion***

Frozen human PBMCs were obtained from Shanghai Public Health Clinical Center. PBMCs were thawed in T cell growth medium (TCM), consisting of X-VIVO 15 medium, human IL-7, human IL-15 and human IL-21, and then allowed to rest for 4-6 h. Before lentiviral transduction, PBMCs were stimulated for 24-36 h with anti-hCD3/hCD28-coated immunobeads at a 1:1 cell:bead ratio in TCM. For transduction, freshly concentrated lentiviral particles were incubated with the activated T cells on NovoNectin (Novoprotein # GMP-CH38)-coated 48-well flat plates at 32°C, mixed with 10 μg/mL protamine sulfate (Sigma #P3369-1OG), and then centrifuged at 1000×g for 1.5 h. The next day, the culture medium was replaced with fresh TCM. At days 6-7 after T cell transduction, the immunobeads were removed, and T cells were expanded until they were rested and could be used in assays. During *ex vivo* expansion, TCM was replenished, and the cell density was adjusted to 0.5-2×10^6^ cells/mL every 2-3 days.

***Generation of the hypoxia-induced reporter system/CAR-modified Jurkat T cells***

Jurkat T cells (Clone E6-1) were lentivirally transduced with the hypoxia-induced mCherry-ODD/CD19/HER2/AXL HiCAR that also contained the reporter gene ZsGreen/EGFP to indicate the transduction efficiency. After viral transduction and expansion, engineered Jurkat T cells expressing the reporter gene ZsGreen/EGFP were gated to evaluate the expression of mCherry-ODD or HiCAR after incubation in cobalt dichloride-mimicking chemical or physical hypoxic conditions mimicked by a mobile CO_2_/O_2_/N_2_ Incubator Chamber (Smator 118, China Innovation Instrument Co., Ltd.) with a BD LSRFortessa flow cytometer.

***Surface immunostaining, fluorescence-activated cell sorting (FACS) and Western blot***

To detect mCherry expression in the cell cytoplasm, cells were collected from tissue culture-treated plates to detect mCherry expression in these gene-modified Jurkat T cells expressing the reporter gene ZsGreen. To detect CAR expression on the cell surface, cells were stained with PE-conjugated anti-DYKDDDDK (Biolegend #637310) in FACS buffer (1× PBS containing 2% FBS) at room temperature for 20 min and washed twice. Cells were resuspended in FACS buffer and assessed using BD LSRFortessa, and all the FACS data were analyzed with FlowJo software v10. To detect total CAR expression in CAR-engineered Jurkat T cells, cell lysates were collected for western blot analysis with anti-FLAG antibody (Sigma #F1804-50UG).

***In vitro assessment of HiCAR-T cell cytotoxicity***

The cytotoxicity of engineered T cells endowed with different CARs was assessed using a firefly luciferase-based cytotoxicity assay. A total of 1×10^4^ target cancer cells were seeded into 96-well black flat-bottom tissue culture plates (Greiner #655090) overnight, and the abovementioned cancer cells were cocultured with CAR-T cells at the indicated E:T (effector:target) ratios in 21% O_2_, CoCl_2_ or 1% O_2_. After 24 h of incubation, all the supernatant was removed, and the viability of the target cancer cells was assessed by quantifying the firefly luciferase intensity using the Luciferase Assay System (Promega, E1501) in a GloMax^®^ 96 reader (Promega, E6521). The following formula was used to determine the normalized cytolytic activity: Cytotoxicity = 100 - (Mean luciferase intensity of CAR-T cells treatment group/Mean luciferase intensity of untranduced T cells treatment group) ×100.

***In vivo antitumor activity of HiCAR-T cells***

The animal protocols used in this study were approved by the institutional animal care and use committee (IACUC) of Shanghai Public Health Clinical Center. Female NOD-Prkdcscid Il2rgtm1/Bcgen (B-NDG) (Biocytogen) mice aged 6-8 weeks were inoculated with 1×10^6^ SKOV3 tumor cells, 2×10^6^ NCI-H292 tumor cells, or 5×10^6^ A549-CD19 tumor cells subcutaneously in the right flank. In A549-CD19 tumor-bearing mice, twelve days after tumor inoculation, 1×10^7^ T cells were injected intravenously into these mice. These T cells were either untransduced (control) or engineered with CD19 CAR or CD19 HiCAR. Tumor size was monitored with calipers every 3 days after T cell transfer. In NCI-H292 tumor-bearing mice, 5×10^6^ T cells were injected intravenously into these mice at day 5 and day 10 post-inoculation. In SKOV3 tumor-bearing mice, 5×10^6^ T cells were injected intravenously at day 10 and day 15 post-inoculation. These T cells were either untransduced (control) or engineered with HER2 CAR or HER2 HiCAR. Tumor size was monitored with calipers every 5 or 10 days after T cell infusion. The tumor volume was calculated using the following formula: V = (length × width^2^)/2.

***Statistical analysis***

All data are presented as the mean ± standard error of the mean (SEM) unless otherwise described. Statistical differences between two groups were determined by a paired Student’s t-test (two-tailed) unless otherwise specified. One-way ANOVA and two-way ANOVA were applied to compare the significant differences among three or more groups. A *P*-value < 0.05 was considered statistically significant. All statistical analyses were performed in Prism 6.01 (GraphPad Prism), and statistical significance was reported as **p* < 0.05, ***p* < 0.01, ****p* < 0.001, *****p* < 0.0001.
